# 

**Published:** 1906-03-03

**Authors:** 


### NOTICE TO CORRESPONDENTS.

All MSS., Letters, Books for Review, and other matters intended for the Editor should be addressed The Editor, "The Hospital," The Hospital Buildings, 28 & 29 Southampton Street, Strand, W.C.

The Editor cannot undertake to return rejected MS., even when accompanied by stamped directed envelope.

### Notice to Advertisers and Subscribers.

All Advertisements, Orders for Copies of The Hospital, and Business Communications should be addressed to **THE MANAGER (Not to the Editor)**, The Hospital, 28 & 29 Southampton Street, Strand London, W.C.

The Cost of Subscription, post free, is as follows:—Per week, 3½d.; per quarter, 3s. 3d.; per half-year, 6s. 6d.; per annum, 13s. Foreign and Colonial:—Per annum, 19s. 6d., payable, in all cases, in advance.

**NOTICE.—Vol. XXXVIII. of "The Hospital" April to September 1905), in handsome cloth binding, will shortly be on sale at the office of this Journal, price 10s. 6d. Cases for binding the Numbers contained in this Volume 2s. Index to Vol. XXXVIII., 3½d., post free.**

**The Monthly Part for March is now ready. Price 1s., by post, 1s. 3d.**

### Medical Appointments and Vacancies.

### APPOINTMENTS.

The following house appointments have been made at St. Thomas's Hospital:—

*Casualty Officers* (from April 1, 1906).—(Senior) G. R. Footner, M.B., B.C.Cantab., M.R.C.S., L.R.C.P.; (Junior) F. R. E. Wright, M.B.Lond., M.R.C.S., L.R.C.P., D.P.H.

*Resident House Physicians.*—F. M. Bulley, M.R.C.S., L.R.C.P., M.A.Cantab.; R. C. Jewesbury, M.A., M.B., B.Ch.Oxon., M.R.C.S., L.R.C.P.; W. O. Meek, M.B., B.S.Lond., M.R.C.S., L.R.C.P. (extension); C. St. A. Coles, M.R.C.S., L.R.C.P. (extension).

*House Physicians to Out-patients.*—M. A. Cassidy, M.A., M.B., B.C.Cantab., M.R.C.S., L.R.C.P.; H. S. Sington, M.R.C.S., L.R.C.P.

*Resident House Surgeons.*—H. T. Gray, B.A., B.C.Cantab., M.R.C.S., L.R.C.P.; A. W. Hooker, M.B., B.S.Lond., M.R.C.S, L.R.C.P.; J. H. Drew, M.B., B.S.Lond., M.R.C.S., L.R.C.P.; H. Falk, B.A., M.B., B.C.Cantab., M.R.C.S., L.R.C.P.

*House Surgeons to Out-patients.*—R. J. H. Cox, M.R.C.S., L.R.C.P.; F. S. Hewett, B.A.Cantab., M.R.C.S., L.R.C.P.; A. B. Howitt, B.A.Cantab., M.R.C.S., L.R.C.P.; W. G. Howarth, M.A., M.B., B.C.Cantab., M.R.C.S., L.R.C.P.

*Obstetric House Physicians.*—(Senior) R. E. Whitting, M.A., M.B., B.C.Cantab.; (Junior) S. R. Gibbs, M.R.C.S., L.R.C.P.

*Ophthalmic House Surgeons.*—(Senior) C. R. B. Eyre, M.R.C.S., L.R.C.P.; (Junior) H. E. Gotelee, M.R.C.S., L.R.C.P.

*Special Departments.* — (Throat) S. G. Macdonald, B.A.Cantab., M.R.C.S., L.R.C.P., and H. B. Whitehouse, M.R.C.S., L.R.C.P.; (Skin) W. O. Sankey, M.R.C.S., L.R.C.P., and A. N. Dickson, M.R.C.S., L.R.C.P.; (Ear) N. R. Cunningham, B.A.Cantab., M.R.C.S., L.R.C.P., and A. L. Loughborough, M.R.C.S., L.R.C.P.

*Children's Surgical.*—C. M. Page, M.R.C.S., L.R.C.P., and S. G. MacDonald, B.A.Cantab., M.R.C.S., L.R.C.P.

*Electrical Department, x-Ray Department.*—N. R. Cunningham, B.A.Cantab., M.R.C.S., L.R.C.P.

### THE NURSE'S BOOKSHELF

*NEW BOOKS NOW READY*

### LECTURES ON THE NURSING OF INFECTIOUS DISEASES.

By F. J. WOOLLACOTT, M.A., M.D.

Senior Assistant Medical Officer, Park Hospital, Lewisham.

Price 2/6 net, 2/9 post free.

### LECTURES ON HOME NURSING FOR THE POOR.

By A DISTRICT NURSE.

A Series of Lectures based upon the observation of what Teaching on Nursing the Poor most need. A very valuable help to all Nurses engaged in District Work.

Price 1/6 net, 1/8 post free.

### NURSING HINTS TO PROBATIONERS ON PRACTICAL WORK.

By ANNESLEY VOYSEY.

Contains full particulars of a Nurse's duties, and will prove of the greatest assistance to the Probationer Nurse.

Price 2/- net, 2/4 post free.

### SURGICAL INSTRUMENTS AND APPLIANCES USED IN OPERATIONS.

By HAROLD BURROWS, M.B. (Lond.), F.R.C.S.

Assistant Surgeon to Bolingbroke Hospital.

An Illustrated and Classified List, with Explanatory Notes.

Price 1/6 net, 1/8 post free.

### THE BURDETT SERIES.

Bound in Cloth Boards, 1/- each, post free.

**PRACTICAL HINTS ON DISTRICT NURSING.**
By Amy Hughes, General Superintendent, Queen Victoria's Jubilee Nurses.

**HOSPITAL AND PRIVATE NURSING.**
By S. S. Orme.

**THE MIDWIVES' POCKET BOOK.**
By Honnor Morten.

**FEVERS AND INFECTIOUS DISEASES.**
By Dr. William Harding.

**MENTAL NURSING.**
By Dr. William Harding, Superintendent Berry Wood Asylum, Northampton.

**SICK NURSING AT HOME.**
By G. Moberly.

**GYNÆCOLOGICAL NURSING.**
By Dr. Hawkins Ambler.

**SYLLABUS OF LECTURES TO NURSES.**
By Dr. Andrew Davidson.

*Complete Catalogue of Nursing Manuals, Case Books, Charts, &c., post free on application.*

### THE SCIENTIFIC PRESS, LTD.,

**28 & 29 Southampton Street, Strand,**

**LONDON**

